# Significant Association Between Huntingtin Gene Mutation and Prevalence of Hopelessness, Depression and Anxiety Symptoms

**DOI:** 10.15388/Amed.2020.28.1.4

**Published:** 2021-01-21

**Authors:** Adelė Butėnaitė, Robertas Strumila, Aistė Lengvenytė, Indrė Kotryna Pakutkaitė, Aušra Morkūnienė, Aušra Matulevičienė, Edgaras Dlugauskas, Algirdas Utkus

**Affiliations:** Psychiatric Clinic, Institute of Clinical Medicine, Faculty of Medicine, Vilnius University, Vilnius, Lithuania; Psychiatric Clinic, Institute of Clinical Medicine, Faculty of Medicine, Vilnius University, Vilnius, Lithuania; Psychiatric Clinic, Institute of Clinical Medicine, Faculty of Medicine, Vilnius University, Vilnius, Lithuania; Vilnius City Mental Health Center, Vilnius, Lithuania; Centre for Medical Genetics, Vilnius University Hospital Santaros Klinikos, Vilnius, Lithuania Department of Human and Medical Genetics, Institute of Biomedical Sciences, Faculty of Medicine, Vilnius University, Vilnius, Lithuania; Centre for Medical Genetics, Vilnius University Hospital Santaros Klinikos, Vilnius, Lithuania Department of Human and Medical Genetics, Institute of Biomedical Sciences, Faculty of Medicine, Vilnius University, Vilnius, Lithuania; Psychiatric Clinic, Institute of Clinical Medicine, Faculty of Medicine, Vilnius University, Vilnius, Lithuania Vilnius University Hospital Santaros Klinikos, Vilnius, Lithuania; Centre for Medical Genetics, Vilnius University Hospital Santaros Klinikos, Vilnius, Lithuania Department of Human and Medical Genetics, Institute of Biomedical Sciences, Faculty of Medicine, Vilnius University, Vilnius, Lithuania

**Keywords:** Huntington’s disease, huntingtin, depression, anxiety, hopelessness

## Abstract

**Summary. Background:**

In Huntington’s disease psychiatric symptoms may manifest prior to motor dysfunction. Such symptoms negatively impact people’s quality of life and can worsen the course of the primary disease. The aim of the present study was to assess and compare depression, anxiety and hopelessness rates in individuals with and without an abnormal expansion of CAG repeats in the huntingtin (HTT) gene and healthy controls.

**Materials and methods:**

Study involved 31 individuals referred for genetic testing for Huntington’s disease and a control group of 41. Depressive and anxiety symptoms were assessed using Beck Hopelessness Scale (BHS) and Hospital Anxiety and Depression Scale (HADS). Results between groups were compared using the Mann–Whitney U test. Two-sided Bonferroni corrected p-value was set at ≤0.017.

**Results:**

Individuals with HTT gene mutation (“gene mutation positive”, GMP) (N=20) scored higher on the HADS depression subscale (5.90 ± 4.52 vs 1.36 ± 1.91; p ≤ 0.017) than those without HTT gene mutation (“gene mutation negative”, GMN) (N=11). GMP and control groups scored higher than the GMN group on the BHS (5.65 ± 3.91 vs 2.09 ± 1.64 and 5.27 ± 4.11 vs 2.09 ± 1.64, respectively; p ≤ 0.017). No differences in anxiety levels were found.

**Conclusions:**

Depressive symptoms and hopelessness were more prevalent in individuals with HTT gene mutation than in individuals who were tested but had no said mutation. Such results emphasise the importance of timely diagnosis and treatment of psychiatric comorbidities in individuals affected by Huntington’s disease.

## Abstract (lt)

**Santrauka. Įvadas:**

sergantiesiems Hantingtono liga psichiatriniai simptomai dažnai pasireiškia anksčiau nei motorinė disfunkcija. Šie simptomai turi neigiam**ą** poveikį gyvenimo kokybei ir gali pabloginti pagrindinės ligos eigą. Darbo tikslas – įvertinti ir palyginti kontrolinės ir asmenų, tirtų dėl huntingtino (HTT) geno mutacijos, grupių depresijos, nerimo ir beviltiškumo simptomatiką.

**Metodai:**

tyrime dalyvavo 31 asmuo, kuriam reikėjo atlikti genetinį ištyrimą dėl Huntingtono ligos, bei kontrolinė grupė, sudaryta iš 41 asmens. Depresijos ir nerimo simptomai buvo vertinami pagal Becko nevilties skalę (angl. *Beck Hopelessness Scale, BHS*) bei Ligonin**ė**s nerimo ir depresijos skal**ę** (angl. *Hospital Anxiety and Depression Scale, HADS*). Rezultatams palyginti taikytas *Mann–Whitney* *U* testas. Pritaikius *Bonferroni* korekciją nustatyta p vertė ≤ 0,017.

**Rezultatai:**

asmenų, kuriems nustatyta HTT geno mutacija (angl. *gene mutation positive*, GMP) (N = 20), HADS depresijos subskalės įverčiai buvo didesni nei asmenų, neturinčių HTT geno mutacijos (*gene mutation negative*, GMN) (N = 11) (5,90 ± 4,52 vs 1,36 ± 1,91; p ≤ 0,017). GMP ir kontrolinės grupių BHS įverčiai buvo didesni nei GMN grupės (5,65 ± 3,91 vs 2,09 ± 1,64 ir 5,27 ± 4,11 vs 2,09 ± 1,64 atitinkamai; p ≤ 0,017). Reikšmingų skirtumų vertinant nerimo simptomatiką nenustatyta.

**Išvados:**

depresijos ir beviltiškumo simptomai buvo dažnesni asmenims, kuriems nustatyta HTT geno mutacija, nei asmenims, kurie buvo tirti, bet šios mutacijos neturėjo. Rezultatai pabrėžia sergančiųjų Huntingtono liga psichikos susirgimų diagnozavimo ir gydymo svarbą.

**Raktažodžiai: **Huntingtono liga, hantingtinas, depresija, nerimas, neviltis.

## Introduction

Huntington’s disease (HD) is a rare neurodegenerative disorder, caused by an expansion of CAG trinucleotide repeats in the huntingtin (HTT) gene, which is located at chromosome 4p16.3 [1,2]. Having from 36 to 39 CAG repeats in one allele of the HTT gene leads to a mutation with reduced penetrance, while at 40 or more CAG repeats it is fully penetrant [3]. Such mutation accounts for the synthesis of the HTT protein with elongated polyglutamine stretch, perturbing normal interactions between proteins, altering their specific functions and ultimately leading to the emergence of HD symptoms [4,5]. 

Initially, neurodegenerative changes are most prominent in basal ganglia, though, as the disease progresses, other parts of the brain, such as the cerebral cortex, the hippocampus, the cerebellum, the hypothalamus and the thalamus, can also be damaged [3,6]. People affected by HD experience an impairment in three main domains – motor, psychiatric and cognitive [7]. Traditionally, the onset of HD is associated with an appearance of motor symptoms, such as coordination issues and involuntary movements. They typically manifest between the age of 35 and 44 years, though in one fifth of the cases motor symptoms can develop before the age of 20 or after the age of 60 [8]. However, psychiatric and cognitive complaints, such as problem-solving difficulties, depression, anxiety and irritability, may be observed many years before the emergence of visible motor dysfunction [9–11]. Indeed, Epping et al. emphasised that individuals at initial stage of HD when motor symptoms are non-visible or mild have the highest prevalence of depression, ranging from 20% to 45% in the asymptomatic and mild symptoms stages, respectively [10]. It is reported that psychiatric symptoms have a greater negative impact on people’s quality of life than motor symptoms or worsening of cognitive functions [12]. Depression can also aggravate chorea [13], accelerate the decline of cognitive functions [14], impair patient functional capacity [15], and is a major suicide risk factor [16]. Since one of the main goals of treating patients with HD is to prevent the decline of their and their family’s quality of life, a timely diagnosis and treatment of comorbid depression may be crucial. Anxiety can also appear in early stages of HD, affecting up to 71% of patients [17], it can be observed separately or as a component of depressive syndrome. While hopelessness is not widely described as an early occurrence in HD, monitoring its levels may be an important component of the suicide risk evaluation [18].

However, studies regarding depressive, anxiety symptomatology and hopelessness in HD patients are lacking. The primary aim of the present study was to assess depression, anxiety and hopelessness rates in individuals referred for genetic testing for the suspected HD. The secondary aim was to compare the scores of those with the confirmed HTT gene mutation, without the HTT gene mutation and the matched controls from the general population. With this study we hope to show that psychiatric comorbidities are common in HD patients, thus timely recognition and adequate treatment of them is crucial. Psychiatric symptoms should not be defined as less important than motor or neurological symptoms, considering their great impact of person’s quality of life. 

## Methods

### Subjects

Study was conducted in Huntington’s Disease Competence Center of Vilnius University Hospital Santaros Klinikos. A total of 72 individuals (N = 31 referred for genetic testing for Huntington’s disease and N = 41 from the general population) were assessed and included in the final analysis during the period between years 2014 and 2015. All patients tested for HD gave the written informed consent. Most of the people referred for genetic testing had relatives affected by HD (N = 29) and experienced motor symptoms (N = 16). In the genetically tested group, an abnormal expansion of CAG trinucleotide repeats was confirmed in 20 people, referred to as a gene mutation positive (GMP) group, whereas it was ruled out in 11 individuals, referred to as a gene mutation negative (GMN) group. 41 matched people from the general population comprised a control group.

### Procedure

Genetic testing for HD patients was performed by polymerase chain reaction (PCR) analysis of the region encompassing the CAG repeat in exon 1 of the huntingtin gene (HTT) followed by fragment sizing through capillary electrophoresis. 

Prior to receiving the results of genetic testing, all participants were assessed for depressive and anxiety symptoms using the Beck Hopelessness Scale (BHS) and the Hospital Anxiety and Depression Scale (HADS).

BHS is a self-administered psychological instrument used in evaluating the extent of individual’s negative attitude (pessimism) about the future. The scale contains 20 questions about major aspects of hopelessness: feelings about the future, loss of motivation and expectations. Respondents score from 0 to 20 points, where higher score corresponds to more intense feelings of hopelessness (0–3 points – minimal, 4–8 – mild, 9–14 – moderate, 15–20 severe hopelessness) [19]. 

HADS is a self-administered instrument designed to evaluate levels of depressive and anxiety symptoms. It contains 14 questions (7 assessing depression symptoms and 7 – anxiety symptoms). In each of the subscales respondents score from 0 to 21 points, where 0–7 points correspond to normal mood, 8–10 – light, 11–14 – medium and 15–21 severe anxiety or depression symptoms [20]. Participants from GMP and GMN groups were also evaluated by a psychiatrist regarding their current and depressive symptomatology and treatment. 

### Data analysis

Quantitative results are presented in means and standard deviations (SD), and qualitative results are expressed in percentages (%). Sociodemographic data was analysed using the Chi-square test. Group comparisons were done using the Mann–Whitney U test. The level of significance was set at two-tailed p≤0.05. Where applicable, the Bonferroni correction was used, and statistically significant differences were stated when p≤0.017. All analyses were conducted with Statistical Package for the Social Sciences 24.0 (SPSS, Chicago, Illinois, USA). 

## Results

**Table 1.** Characteristics of participants.

	**GMP group N (%) or mean ± SD**	**GMN group N (%) or mean ± SD**	**Control group N (%) or mean ± SD**	**Total N (%) or mean ± SD**
Female gender	12 (60%)	4 (36.4%)	22 (53.7%)	38 (52.8%)
Age	47,45 ± 14.29	32,36 ± 10.64	48,56 ± 12.23	45,32 ± 13.91
History of depression	13 (65%)	0 (0%)	1 (2.43%)	14 (19.44%)
HADS-D score	5.90 ± 4.52	1.36 ± 1.91	3.10 ± 2.48	3.61 ± 3.44
HADS-A score	8.00 ± 5.23	4.73 ± 2.61	5.12 ± 3.55	5.86 ± 4.15
BHS score	5.65 ± 3.91	2.09 ± 1.64	5.27 ± 4.11	4.89 ± 3.93

Basic characteristics of participants are presented in Table 1. The present study included 38 (52.8%) female and 34 (47.2%) male participants, and there were no significant gender-based differences between the groups (p > 0.05). There were no significant age differences between GMP and control groups. Participants from GMN group were significantly younger than people from GMP group (32,36 ± 10.64 vs 47,45 ± 14.29; p = 0.006) and healthy controls (32,36 ± 10.64 vs 48,56 ± 12.23; p = 0.001). Ten participants from GMP group had been diagnosed with depressive episodes prior to this study and were already taking antidepressants. During this study, three participants from GMP group were newly diagnosed with depressive episode. The average HAD Depression sub-scale scores in all three groups corresponded to normal mood. The average HAD Anxiety subscale score in GMP group was suggestive of mild anxiety, while average HAD Anxiety subscale scores in GMN and control groups were normal. Finally, the average BHS scores in GMP and control groups corresponded to mild levels of hopelessness, while average score of GMN group showed minimal hopelessness.

**Figure 1.**
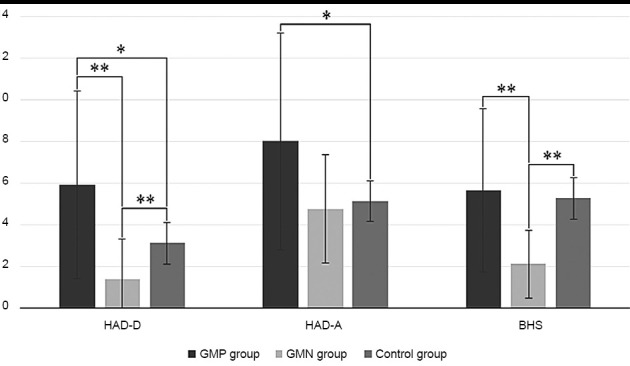
Differences in depressive, anxiety and hopelessness symptomatology between huntingtin gene mutation-positive, huntingtin gene mutation-negative individuals and healthy controls. * p≤0.05, ** Bonferroni correction, p≤0.017

The GMP group had significantly higher HADS-D scores than GMN group (5.90 ± 4.52 vs 1.36 ± 1.91; p = 0.003). There was a tendency for GMP to have higher HADS-D score than healthy controls as well, but this difference did not survive the Bonferroni correction (5.90 ± 4.52 vs 3.10 ± 2.48; p = 0.036). Meanwhile, healthy controls had significantly higher score than GMN (3.10 ± 2.48 vs 1.36 ± 1.91; p = 0.015). GMP group had nominally higher HAD-A score than controls, but this difference did not survive the Bonferroni correction (8.00 ± 5.23 vs 5.12 ± 3.55; p = 0.039). No other differences regarding HAD-A scores were observed. Both GMP and control groups scored significantly higher than the GMN group on the BHS (5.65 ± 3.91 vs 2.09 ± 1.64; p = 0.007, and 5.27 ± 4.11 vs 2.09 ± 1.64; p = 0.007, respectively). Differences between groups in HADS and BHS average scores are presented in Figure 1.

## Discussion

Individuals with chronic medical conditions seem to be particularly affected by comorbid depression [9] and HD is not an exception, with a reported depression rate of more than 40% in a previous study by Epping et al. [10] Findings from the present study are consistent with this trend. Notably, while average scores in HADS depression subscale were suggestive of normal mood in all of the groups, 50% of the patients in GMP group were diagnosed with depression prior to this study and were already taking antidepressants, whereas rates of depression were lower in other groups – no one from GMN group and only 2 individuals (4.88%) from control group had a history of depression. Depressive symptomatology levels in GMP group suggest that antidepressants are effective in treating comorbid depression in HD, though additional studies are required for definitive conclusions. However, despite high rates of ongoing treatment, patients in the GMP group still had higher levels of subclinical depressive symptomatology, which has been shown to be associated with worse functioning in normothymic individuals with mood disorders [21].

There are several possible explanations why people with HTT gene mutation have more depressive symptoms than population without the said mutation. First, depression can develop as a result of brain structural damage and alteration of normal neurophysiology [5,11,22]. HD is caused by an abnormal expansion of polyglutamine (polyQ) in the N-terminal part of HTT protein. When mutated polyQ-HTT protein is broken down by proteases, N‐terminal fragments with an expanded polyQ sequence are released [5,23,24]. Such fragments are neurotoxic – they aggregate and accumulate in cells, leading to neuronal damage [5,11,22,25]. 

Multiple bodily systems are dysregulated in HD and may confer to increased risk of depression [26]. Serotonergic neurotransmission has been reported to be impaired in patients with HD. Indeed, there are reports on a positive correlation between mesencephalic raphe echogenicity and depressive state [27], abnormally high levels of MAO-A activity in basal ganglia and pons [28], and a reduction of 5-HT3 receptor recognition site levels in basal nuclei in patients with HD [29]. The dysregulation of hypothalamic-pituitary-adrenal axis (HPA), often associated with mood and anxiety disorders [30–32], has also been described in HD. Aziz et al. described that damage to suprachiasmatic and paraventricular hypothalamus nuclei disturbs central glucocorticoid feedback loop in HD patients, leading to cortisol hyper-production, possibly contributing to increased risk of depression [33]. Structural and functional changes in such brain regions as the prefrontal cortex, the cingulate cortex, the striatum, the amygdala, the hypothalamus and the thalamus, can also play a role in development of mood disorders in HD patients [11,34]. Present study did not investigate neurobiological changes in individuals affected by HD, though this could be assessed in future studies. While a direct damage to different brain structures is considered to be the main etiological factor of depression in HD patients, the role of psychosocial factors should not be excluded. HD is a rapidly progressing illness – patients experience a dramatic worsening of symptoms, loss of functional capacity and have to constantly re-adjust to the new role in the social setting, which perturb their emotional state [7]. 

Assessing depression in patients suffering from HD can be complicated – there’s a risk for depressive symptoms to be mislabelled as a “normal” psychological reaction to the disease and its progression or, conversely, HD symptoms can be mistakenly attributed to a mood disorder. Progressive cognitive impairment and communication issues in later stages of HD may further contribute to diagnostic challenges [10,35]. Notably, apathy, frequently associated with HD, can also be mislabelled as depression in HD patients and vice versa. Both have many similarities, but apathy is mostly recognised as an impairment in person’s motivation and goal-oriented behaviour, whereas depression typically has a stronger emphasis on emotional dimension [36]. Naarding et al. reported that apathy was more common in later stages of the HD and was linked to cognitive deterioration and functional decline, while depression was usually an early occurrence in HD [37]. While these differences may seem negligible, it is important to differentiate these neuropsychiatric syndromes due to their distinct treatment approaches. While depression is conventionally treated with serotonergic drugs, patients with apathy as a primary complaint may respond positively to psychostimulants, which act on dopamine system [8].

Alongside depressive symptoms, we also evaluated anxiety and hopelessness. Anxiety and hopelessness are common components of depressive syndrome and can increase the risk of suicide [18,38]. In the present study, participants with an abnormal CAG repeats in the HTT gene expressed significantly higher hopelessness than those who received genetic testing but did not have such abnormalities in the said gene. Interestingly, both the control and GMP groups showed mild hopelessness levels. Such hopelessness score in the control group, as well as their relatively high scores in HADS depression subscale, may be influenced by the fact that control group included only medical personnel from two hospitals (control group was formed using the convenience sampling method). Numerous studies have reported high rates of depression as well as burnout syndrome in physicians, which are conditions associated with increased levels of hopelessness [39–41]. In future studies, it would be beneficial to select a random sample to have a better representation of the general population. Nevertheless, the GMP group had higher hopelessness than the GMN group and this difference is not attributable to a feeling of relief, considering that psychological tests were applied prior to receiving the results of genetic testing. 

Furthermore, though not significantly different from other groups, increased level of anxiety was observed in 35% of participants from GMP group, in line with previous report by Dale et al., which found that the prevalence of anxiety in HD ranged from 13% to 71% [17]. 

Notably, suicide accounts for up to 10% of all deaths in this population [42], making HD the neurodegenerative disorder with the highest suicide risk [43]. Starting from the early stages, depression, anxiety and loss of functional capacity have been reported to contribute to increased rates of suicidal behaviours in HD patients [44,45], underlining the importance of early detection and intervention in these conditions.

## Limitations

The present study has several major limitations. A small sample size is inherent to a rarity of the Huntington’s disease. This issue could be addressed by conducting multicentre studies or extending the period of recruitment in the future. Another limitation is that the control group in the present study was formed by the convenience sampling method and included only medical personnel from two hospitals. Owing to high burnout rates in the medical population, this could have led to higher scores in HADS and BHS scales and less differences compared to other groups. In future studies, we suggest random sampling of control group to better represent the general population. Furthermore, 10 participants with huntingtin gene mutation were diagnosed with depression and received treatment prior to this study, and the effect of drugs on their symptoms could not be assessed. Despite this, these individuals still presented with higher rates of subclinical depression. Together, these findings suggest that depression is often comorbid in HD and manifests early in the course of the disease. Though this study was naturalistic and reflecting real clinical practice, when patients are often already treated when they are referred to genetic testing, in future, it would be beneficial to exclude people already taking antidepressants from the study and assess depressive symptoms before any psychopharmacological intervention.

## Conclusions

In sum, the present study adds to the evidence that depression, anxiety and hopelessness are common in patients suffering from HD already in early stages. Individuals with an abnormal expansion of CAG repeats in HTT gene had more depressive symptomatology than those who were also tested, but did not have the said abnormalities. Future studies should assess the effective interventions to address mood and anxiety symptoms in early stages of HD. Results emphasize the importance of timely treatment of comorbid depression and anxiety disorders.

## Conflict of interest

The authors declare that they have no conflict of interest.

## Abbreviations

HADS = Hospital Anxiety and Depression Scale

HAD-D = HADS Depression subscale

HAD-A = HADS Anxiety subscale

BHS = Beck Hopelessness scale

GMP group = huntingtin gene mutation positive group

GMN group = huntingtin gene mutation negative group.
